# Appropriate and acceptable health assessments for people experiencing homelessness

**DOI:** 10.1186/s12889-022-13723-7

**Published:** 2022-07-04

**Authors:** Susan Jayne Gordon, Nicky Baker, Margie Steffens

**Affiliations:** 1 College of Nursing and Health Sciences, Flinders University, Bedford Park, South Australia 5042 Australia; 2 Director Community Outreach Dental Program, University of Adelaide, Adelaide, South Australia 5000 Australia

**Keywords:** Homeless persons, Feasibility, Health status, Vulnerable populations

## Abstract

**Background:**

Appropriate and acceptable recruitment strategies and assessment tools are essential to determine the health needs for people experiencing homelessness. Based on a systematic review and known feasible community-based health assessments for people who are not homeless, a set of health assessments were trialled with people experiencing homelessness.

**Methods:**

Participants were recruited via support agencies. They completed a health risk assessment, demographic and self-report health questionnaires, and objective assessments across 17 domains of health.

**Results:**

Fifty-three participants (43.3% female, mean age 49.1 years) consented and completed 83–96% of assessments. Consent was reversed for assessments of grip, foot sensation, body measures (11%), and walking (30%), and initially refused for stress, sleep, cognition (6%); balance, walk test (9%) and oral examination (11%). There was one adverse event. Most assessments were both appropriate and acceptable. Some required modification for the context of homelessness, in particular the K10 was over-familiar to participants resulting in memorised responses. Recruitment strategies and practices must increase trust and ensure participants feel safe.

**Conclusions:**

This set of health assessments are appropriate and acceptable for administration with people experiencing homelessness. Outcomes of these assessments are essential to inform public and primary health service priorities to improve the health of people experiencing homelessness**.**

## Background

According to the Australian Bureau of Statistics, people are considered to be homeless “if their current living arrangement: is in a dwelling that is inadequate; or has no tenure, or if their initial tenure is short and not extendable; or does not allow them to have control of, or access to space for social relations” ([1] , p.7). Homelessness results from complex, inter-related and cascading events, for instance domestic violence, no or inadequate income, illiteracy, unemployment, physical and/or mental health concerns, and/or consequences of lifestyle issues such as gambling, alcohol or drug addiction [2]. It is well-established internationally that an initial event of homelessness can become a recurring cycle from which individuals find it difficult to escape [3].

Homelessness has significant impacts on the health and health-seeking behaviours of people who experience it. It places significant burdens on individuals’ physical and mental health [3] which, in addition to reducing access to medication, healthcare, nutrition, employment and education, can lead to premature ageing [2]. Although not necessarily the cause of homelessness, addiction may also be associated with an altered ageing process among people who experience homelessness [4]. An additional consequence of reduced access to medication and healthcare is that many adults experiencing homelessness are frequent users of acute healthcare services such as emergency departments [5]. Many do not, or cannot, access preventative or supportive healthcare services.

Utilisation of health care resources requires individuals to perceive they have access to health care systems, and this perception of accessibility can be mediated by characteristics of the users and providers of health services [6]. Levesque et al., (2013) developed a framework to conceptualise five key determinants of user accessibility of healthcare: 1) approachability, 2) acceptability, 3) availability and accommodation, 4) affordability and 5) appropriateness. Acceptability and appropriateness are interrelated dimensions that can be considered beyond the scope of access due to geography, service availability and affordability, as it encompasses considerations of individuals’ opportunity to make informed choices about their participation in their own care and be empowered in decision-making and self-management. Ensuring that services meet the cultural values, norms, and expectations of population served, and provide adequate quality care that effectively addresses their health needs is essential to providing acceptable and appropriate care [6].

A range of policy recommendations have been made to improve the health of homeless populations. These include training staff of homeless shelters to identify the healthcare needs of homeless populations [7] by including geriatric services [8] and supplying coordinated and comprehensive healthcare services at pre-existing housing services [9]. These recommendations share common themes of providing easily accessible healthcare services tailored to the health needs of its users. However, the recommendations assume the health screening tools meet several criteria including can be undertaken in diverse settings; are administered competently by health professionals of varying backgrounds; and are acceptable to people experiencing homelessness. The health needs of end-users must be well understood to effectively design, implement and evaluate these recommendations. This requires research to determine appropriate health measures and health outcomes for this population.

People experiencing homelessness can be vulnerable in the research process as the focus of investigations can be mounted from a position of unequal power and pre-conceived researcher perspectives [10]. Consequently, research investigations may impose unrecognised burdens on potential participants, who may lack the capacity, literacy, insights and/or confidence to refuse [11]. There are ethical requirements surrounding the capture of information from vulnerable populations, including recognition and prevention of coercion to consent, fatigue from over-research, and perceived pressure to disclose sensitive information, or to participate in activities that may cause distress. There are also ethical considerations regarding the safety of researchers, including prevention of physical and psychological harm [12].

Understanding the health needs of people experiencing homelessness poses methodological challenges which have been recognised for over 20 years [13, 14]. It specifically requires the use of end-user-oriented strategies which transparently demonstrate beneficence and non-malfeasance, thereby optimising recruitment and retention.

This paper reports a trial of general health assessments conducted with people experiencing homelessness. It reports the outcomes of the recruitment strategies used, the appropriateness and acceptability of the assessments administered based on the rate of participation in the health assessments, occurrence of adverse events, and participants’ and health assessors’ feedback regarding their perspectives on the research approach.

## Materials and methods

### Ethics

Ethics approval was provided from the Southern Adelaide Clinical Human Research Ethics Committee (222.17).

### Design

Cross-sectional, observational study.

### Recruitment

One successful recruitment strategy for people experiencing homelessness is to partner with organisations that are trusted and frequented by people experiencing homelessness; where people feel safe, supported, and empowered to exercise their choices. Thus, this research identified a key partner: Common Ground, an organisation under the auspices of Housing Choices, South Australia. Common Ground is an internationally established and well-known local organisation that provides accommodation for people who are homeless and those at risk of homelessness. In addition to providing low cost, affordable accommodation they offer dental care and some limited medical services to their tenants. Beyond the benefit for individual participants in this research Common Ground identified the benefit from a health needs analysis to inform future health services planning. Secondary partners were other organisations in inner city Adelaide providing services to people experiencing homelessness (hostels and shelters). Recruitment used a convenience snowballing approach, with participants alerted to the project by posters, direct invitation by Common Ground staff and word-of-mouth.

Potentially eligible participants were invited to attend Common Ground during the test period, to share food and observe data collection. They could view activities, ask questions, and choose whether to participate in testing once they understood its purpose and processes. Eligible participants were aged 18 years or older and met the ABS definition of homelessness. There were no exclusion criteria.

### Ethical considerations

To ensure participants were fully informed and supported, organisation staff independent of the research team were invited to accompany potential participants, to explain the project to those with hearing or vision difficulties, and support those who were anxious about participation. Researchers described all assessments to participants and their supporting staff member in as much detail as was required for the potential participant to understand and willingly provide informed consent for each assessment activity. Participants confirmed consent at each testing station. When an assessor identified risk, participants were counselled not to consent to the test. During assessment, participants’ health and willingness to take part in the research were regularly monitored, and they were reminded at each testing station that they could refuse to participate in any assessments or withdraw at any time from the project.

### Selection of health assessments

A systematic review helped to identify health screening and assessment tools specifically developed for, validated with, and/or administered to, populations experiencing homelessness [15]. Two assessments that had previously been successfully administered with populations experiencing homelessness were selected from this review. However, neither had been developed for or validated with people experiencing homeless. Three additional activities were identified from the health assessments conducted in the Inspiring Health project, a community-based health screening service assessing the general health of community-dwelling adults aged 40–75 years [16]. Common measures that are relevant to any adult irrespective of living circumstances (e.g., blood pressure, heart rate, and spirometry) were also included, and additional activities and data items were identified in consultation with staff and health professionals working at Common Ground. Alterations were made to some wording to ensure that assessments “use [d] language, items and constructs which are relevant to homelessness” ([15] , p.2). The final assessments used are listed and referenced in Table 1.

**Table 1:** Station-based health assessments for people experiencing homelessness. The **bolded measures** are relevant for administration to a homeless population. The ***bolded italic*** measures were developed for, or validated on, homeless populations. The underlined measures have validated thresholds for comparison across samples. (Cronbach’s alpha reported for scales where known)

**Health risk screen to establish ‘safe to participate’ in the health screening activities** *Self report Objective* (NHNS 2017 [17]; NHS 2007 [18])	
Pain at time of assessment Known medical conditions **Medications**	**Blood pressure** (thresholds available) **Blood oxygenation** (thresholds available) **Heart rate** (thresholds available) **Respiration rate** (thresholds available) **Temperature** (thresholds available)
**1. Survey: Socio-demographic information** *(Self-report with or without literacy assistance)*	
**Age** **Gender** **Culture & ethnicity** **Language** **Marital status** **Falls and near misses (past 6 months)** **Vaccination history (past 5 years)**	**Living arrangements** **Education** **Employment** **Pet ownership** **Most common form of transport used** **N. unplanned admissions to general medical practitioners, hospital emergency departments (ED) or hospital wards in past 12 months** **Health screenings & vaccinations in last 12 months**
**2. Survey: Self-reported health** *(Self-report)*	
***DETERMINE your Nutritional Health*** [19] (α 0.445, [20]) **Pelvic Floor Bother Questionnaire** (PFBQ) (α = 0.61, [21])	**Clinical Frailty Scale** (CFS [22];)
**3. Objective health assessments**	
***Station 1: Cognition*** *(Psychologist / Social worker)*	***Station 5: Dexterity, response time*** *(Physiotherapist)*
**General Practitioner Assessment of Cognition** **(GPCog)** (α = 0.84, [23]) **Kessler Psychological Distress Scale** (K10) [24] **(**α = 0.93) [25] **Pittsburgh Sleep Quality Index** **(PSQI)** (α = 0.83, [26])	**Purdue Dexterity Test** **(PDT) (**[27]) **Ruler reaction time** [28]
***Station 2: Audiometry*** *(Audiologist)*	***Station 6: Mobility & exertion*** *(Physiotherapist)*
Otoscopy ( [29] **Audiometry** [29] **Speech, Spatial and Qualities of Hearing Questionnaire** **(SSQ5**) [30]	Balance Screening Tool (BST, [31]) **Six-minute Walk Test** (SMWT, [32]) **Rating of Perceived Exertion Scale** and **Dyspnoea scale** [33]
***Station 3: Functional movement & grip strength*** *(Physiotherapist)*	***Station 7: Skin, eye and foot health*** *(General practitioner)*
Functional Movement Screen [34] **Grip strength** [32]	**Skin check** [17] **Eye health & vision test** [17] **Foot sensation** [35]
***Station 4: Lung function & anthropometry*** *(Respiratory scientist & physiotherapist)*	***Station 8: Oral health*** *(Oral hygienist with dental students)*
**Lung function: spirometry** [36] **Body Mass Index (BMI)** [17] **Waist and hip circumference** [17]	***Oral Health Impact Profile– 14*** (OHIP-14, [37]) **ARCPOH Oral Health Questionnaire** including mouth and teeth health [38]

### Data collection

Health assessments were administered over six days in November and December 2017 at Common Ground, Adelaide. Data were collected by survey and by objective measurement conducted by the assessors: eight experienced healthcare professionals (see Table 1) who were recruited using advertisements emailed to the networks of Common Ground and XXX. An initial risk assessment of health and physiological measures was used to establish fitness to participate. It included blood pressure, temperature, heart rate, presence of pain and general health. If anyone was considered at risk of adverse outcomes from participation, they were offered medical attention and to take no further part in data collection. Based on the outcomes of the risk assessment, modifications were made to subsequent assessments as required to accommodate people with injuries or disability.

Following the risk assessment, participants were randomly assigned to their first measurement station and then progressed to subsequent assessment stations. If risk of harm was perceived, the participant moved to the next measurement station, and thus proceeded sequentially until all assessments were completed or they withdrew. The full assessment procedure occurred in one 150-minute session.

### Acknowledgement of participation

All participants were invited to a shared lunch either before or after their assessment. They were also provided with a selection of oral and personal hygiene products to acknowledge their time in participating.

### Data analysis

#### Appropriateness, acceptability and participation in health assessments

This was captured in three ways:

- The willingness of participants to undertake assessments (acceptability), and rates of completion (appropriateness) of each test. Comments were recorded including issues experienced when attempting or completing tests, reluctance to participate in any or all activities, reasons for reluctance or refusal if offered, and any comments on the tests***.***
- Assessors counselling participants not to complete an assessment based on their medical history and/or risk assessment conducted on the day.
- Adverse events. These were any unexpected event or distress, such as pain from participating in physical assessment, loss of balance (near fall or fall) or distress when responding to questions.

#### Feedback from assessors

Written and verbal feedback was captured as specific events of concern, anecdotes offered during debriefing sessions conducted every day, or health issues that were experienced during data collection. This was done in conjunction with staff from the partner agency Common Ground who were experienced in working with the participants and could provide insights into behaviours, triggers and ways of coping. Semi-structured interview questions were used in each debriefing session.

### Data management

Participant data were de-identified. Refusal to participate, assessor advice not to attempt or complete, unsuccessful attempts at health assessments, and completed health assessments were captured per participant in a purpose-built MS Excel spreadsheet. Adverse events for researchers and participants, and participant commentaries were captured as structured observer notes throughout data collection. Researcher feedback was noted during post-data collection debriefing sessions.

Observer notes collected from the assessors and researcher feedback noted during the debriefing sessions were collated and coded by the authors. A comprehensive summary of participant behaviours, concerns and experiences was developed through discussion between the researchers and assessors.

## Results

Fifty-three participants ranging from 22 to 84 years of age provided informed consent and participated in at least 50% of the assessments. Participants comprised 23 women (43.4%) with a mean age of 49.1 years. The majority were living in supported accommodation (76%) or a rented room (16%) as per clause (b) of the homelessness definition: having no tenure, or short and not-extendable tenure. All participants had previously experienced living in a dwelling that was inadequate or did not allow them to have control of, and access to, space for social relations, as per clauses (a) and (c) of the homelessness definition. Participants’ demographic data is described in Table 2.

**Table 2:** Demographic data by gender

	Male		Female		Overall	
	n	%	n	%	N	%
**Culture and ethnicity (*n*** **= 52)**						
Australian	20	69.0	14	60.9	34	65.4
British	3	10.3	3	13.0	6	11.5
European	3	10.3	3	13.0	6	11.5
Other (Middle East, Indian, PNG)	3	10.3	3	13.0	6	11.5
**Main language spoken (*n* = 51)**						
English	27	96.4	20	87.0	47	92.2
Other	1	3.6	3	13.0	4	7.8
**Living arrangements in the last week (*n* = 50)**						
Outside/Shelter/Other	–	–	4	17.4	4	8.0
Rented room	4	14.8	4	17.4	8	16.0
Supported accommodation	23	85.2	15	65.2	38	76.0
**Shared living space in the last week (*n* = 50)**						
Yes	12	42.9	14	63.6	26	52.0
No	16	57.1	8	36.4	24	48.0
**Marital status (*n* = 52)**						
Married/de facto	2	6.9	2	8.7	4	7.7
Separated/divorced/widowed	9	31.0	4	17.4	13	25.0
Single	18	62.1	17	73.9	35	67.3
**Income (*n* = 52)**						
Pension	26	89.7	22	95.7	48	92.3
Wage	2	6.9	1	4.3	3	5.8
Other	1	3.4	–	–	1	1.9
**Main means of transport (*n* = 51)**						
Walk	8	28.6	9	39.1	17	33.3
Bicycle	3	10.7	1	4.3	4	7.8
Car	3	10.7	4	17.4	7	13.7
Public transport	14	50.0	9	39.1	23	45.1

### Appropriateness and acceptability

On average, 88% of participants completed each station-based assessment. The six-minute walk test (SMWT) had the lowest rate of completion, with approximately 50% of participants consenting to and completing the task. However, approximately one quarter of participants who did not complete the SMWT were counselled not to attempt the assessment due to adverse weather conditions for the outside activity (rain, or temperatures exceeding 40 degrees Celsius). Excepting the SMWT and the health risk screen (which all participants were required to undertake), assessment completion rate ranged between 83 and 96%.

**Fig. 1:**
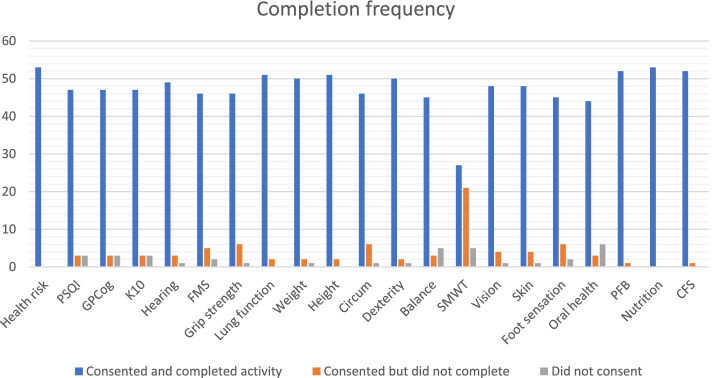
Completion numbers for each assessment (N)

At baseline, nearly 6 % of participants did not give consent to participate in the stress, sleep and cognition assessments. Nine percent did not give consent to participate in the balance (BST) or SWMT, and a further 11 % declined consent for the oral examination and questionnaire. Between four and 30 % of participants reversed their consent at specific measurement stations (i.e., provided consent at baseline but elected not to complete the assessment when they reached that station: see Fig. 1). Thirty percent of participants reversed their consent for the SMWT, and 11% reversed their consent for grip strength, foot sensation and anthropometry (height, weight, waist and hip circumference).

There was one adverse event. This occurred to a 45-year-old female participant who stumbled during a dynamic movement test and reported pain as a result. The participant lodged a complaint with the researchers who reported this to the ethics committee. She also expressed concerns that she had been coerced to participate, and that her right to choose had not been respected (despite documented assurances to the contrary). This resulted in early cessation of recruitment and data collection. There were no reported violations of assessment procedures at any measurement station, and no participant or researcher complaints about how assessments were conducted.

### Feedback from the assessors

The assessors valued the daily debriefs as, despite their professional experience, most reported being confronted by unexpected events at the facility and the traumatic events and histories shared by participants during their assessments. They also recognised the high levels of anxiety and exaggerated responses from participants, especially at commencement of the assessments. Assessors described specific events of concern including: being confronted by aggressive behaviour; contextual normalisation of drug- and alcohol-related behaviours; the high level of suspicion by potentially-eligible participants about the research and the researchers; difficulties of recruiting and then gaining informed consent, including researchers’ heightened awareness of the potential for their behaviours to be perceived as coercion; and the speed with which participants lost interest in the project throughout all stages of recruitment, consent and participation. Assessors also reported their own health adverse events (albeit minor), including skin infections and a case of parasite infestation.

## Discussion

The health assessments applied in this study were appropriate and acceptable for most participants. Future researchers can use them confidently knowing that they are acceptable to assess health status of people experiencing homelessness. However, many lessons were learnt to improve recruitment and engagement of participants and support for assessors.

There was suspicion about the motives of researchers, even those members of the research team known to the participants. The level of suspicion in potential and actual participants encompassed many issues, such as the threat of eviction from current accommodation, fear of withdrawal of access to health and social support services if people did not participate, the fear of being diagnosed with a disease, and concerns about general disclosure of their health problems. There also appeared to be a power differential: participants could see that the partner agency valued the research and thus some participants indicated informally to the assessors that they were placed in a potentially vulnerable situation if they did not agree to provide data that the agency could use. Independent recruitment might overcome this but would equally remove participants from a setting that was familiar and presumably felt safe.

The snowballing recruitment strategy appeared to be effective in attracting increasing numbers of interested and consenting participants over the data collection period as the word spread about the research intent and process. Being supported by staff from their usual housing agency appeared to be helpful, particularly for people who were anxious or sick, or had vision, hearing or mobility problems.

Providing a free meal was a critical element in recruitment. It provided an acceptable way for people to attend and observe, irrespective whether they committed to participate on the day. Several people attended to eat with the assessors and other participants before agreeing to participate; others came to eat and only observe.

It had originally been considered a positive strategy to engage accommodation agencies as partners because it built on previous relationships with participants. However, the fragility of this relationship was underestimated, particularly when participants had mental health issues, or had a history of housing difficulties such as eviction. The potential for perceived coercion or unauthorised use of data by the partner agencies was high, despite assurances that participants could withdraw at any time and safeguards such as deidentification of data being in place. The assessors concluded that data collection in this context required experienced and emotionally mature staff. Collecting data from people experiencing homelessness would be an unsuitable, and even a potentially damaging experience, for students or inexperienced researchers and health professionals without significant support.

No participant’s health was sufficiently poor to prevent them participating in the project. The participants who consented to undertake assessments appeared to be satisfied with the activities that occurred at each measurement station and their experience in the project generally. The assessments appeared to be acceptable to most people.

Researchers noted that most participants were familiar with the K10 Psychological Distress Scale. Many could recite the questions, knew the high and low score range and what this meant for scoring their mental health. It appeared from participant feedback that this instrument was regularly administered during their health checks, and this familiarity therefore may make it an invalid tool in this population, because it creates pre-established response bias [39].

To minimise the burden on participants, the range of assessments included was limited to those considered to be the most essential and accurate for populations of people experiencing homeless. Additionally, some assessments that may have been considered particularly invasive were excluded, to avoid discouraging individuals from participating. Some important aspects of health, such as the reproductive history and health of women experiencing homelessness, were not assessed, but may be valuable inclusions for future investigations.

In this city, there was no central agency from which individuals could be comprehensively recruited. People experiencing homelessness rarely have a cohesive community voice, and therefore any sample will potentially have threats to the external validity of its findings. In a vulnerable population such as those who have experienced, and are experiencing, homelessness, the fragility of relationships with housing agencies supporting them, and their perceptions regarding lack of trust, respect and choice cannot be under-estimated. Even though the recruitment and data collection methods for this study supported individual choice at every stage and provided agency staff support for participants, it appeared some participants did not fully appreciate their rights, or their capacity to exercise them. Alternative recruitment strategies may be more effective, such as sourcing people from food distribution agencies or hostels, where there is less opportunity for perceived coercion.

This study captured comprehensive health data to establish priority health needs in this population. The number of assessments administered may be decreased once there is better understanding of the health priorities. This would reduce the significant time impost (2.5 hours) of the current study. People experiencing homelessness are busy. Daily tasks such as attending social services and sourcing food by foot or public transport takes a large part of the day. Participant fatigue and distractibility was noted and future data collection should aim to prioritise health assessments so that key measures are captured early in the test period as a priority. Any additional and perhaps less important measures need to be questioned as to their relevance, but if deemed necessary could be captured when participants were fatiguing.

Familiarity with tests such as K10 highlighted the potential for under- or over-reporting of mental health problems. The challenges of assessing mobility using the SMWT in outdoor spaces were highlighted. Future data collection using this test should access an indoor venue where weather is not a concern, or alternatively use another test.

The importance of spending time building relationships between researchers, assessors, clinicians, participants and agency staff cannot be overstated. This could have been improved by letting potential participants observe a pilot data collection session so that they could become familiar with the process and comfortable with their involvement in it. An important learning was the provision of meals. This ensured that participants received a tangible benefit from being interested in the research. Not only was this an opportunity for participants to congregate in the test venue, but it provided a socially acceptable way for them to observe data collection without the need to participate. Participants’ lack of trust in the research process potentially relates to previous poor experiences when interacting with strangers and healthcare professionals. Thus, recruitment strategies should recognise these vulnerabilities and work around them in innovative ways to ensure that people experiencing homelessness are more equal partners in the research.

A key learning was the importance of the researchers’ and assessors’ debriefing at the end of the day. The engagement of Common Ground staff in this process provided helpful insights and enabled researchers to put their concerns into perspective. It was also clear that less experienced researchers, clinicians, and students, should not be engaged in projects such as this, because of the many and unforeseen challenges of collecting data from this vulnerable population.

Understanding the health and social needs of vulnerable people experiencing homelessness is essential to underpin evidence-informed policy. From our experiences, the health assessments included were acceptable. Recruitment and engagement strategies for participants and support for assessors are suggested. Further studies would allow identification of a minimum number of health assessment that will provide enough information on health needs without threatening beneficence and non-malfeasance.

## Data Availability

The datasets generated and analysed during the current study are not publicly available due to the sensitivity of the data collected. Data may be available from the corresponding author [SG] upon reasonable request.
